# RETRACTED: Abdi et al. Duloxetine, an SNRI, Targets pSTAT3 Signaling: *In*-Silico, RNA-Seq and *In*-Vitro Evidence for a Pleiotropic Mechanism of Pain Relief. *Int. J. Mol. Sci.* 2025, *26*, 10432

**DOI:** 10.3390/ijms27135656

**Published:** 2026-06-23

**Authors:** Sayed Aliul Hasan Abdi, Gohar Azhar, Xiaomin Zhang, Jeanne Y. Wei

**Affiliations:** Department of Geriatrics, Donald W. Reynolds Institute on Aging, University of Arkansas for Medical Sciences, Little Rock, AR 72205, USA

The journal retracts the article titled “Duloxetine, an SNRI, Targets pSTAT3 Signaling: *In*-Silico, RNA-Seq and *In*-Vitro Evidence for a Pleiotropic Mechanism of Pain Relief” [1], cited above.

Following publication, concerns were brought to the attention of the Editorial Office regarding data and scientific issues in this publication [1].

In accordance with standard journal procedures, the Editorial Office and Editorial Board conducted an investigation that confirmed inconsistencies in the molecular docking simulations and identified several methodological limitations in this article [1]. Although the authors collaborated with the investigation and shared supporting materials, the Editorial Board concluded that the evidence presented was insufficient to support the article’s overall findings and conclusions. As a result, the Editorial Board has decided to retract this paper as per MDPI’s retraction policy.

This retraction was approved by the Editor-in-Chief of the journal *IJMS*.

The authors have been informed of this retraction and have indicated their disagreement with this decision.
